# LHX2 Interacts with the NuRD Complex and Regulates Cortical Neuron Subtype Determinants *Fezf2* and *Sox11*

**DOI:** 10.1523/JNEUROSCI.2836-16.2016

**Published:** 2017-01-04

**Authors:** Bhavana Muralidharan, Zeba Khatri, Upasana Maheshwari, Ritika Gupta, Basabdatta Roy, Saurabh J. Pradhan, Krishanpal Karmodiya, Hari Padmanabhan, Ashwin S. Shetty, Chinthapalli Balaji, Ullas Kolthur-Seetharam, Jeffrey D. Macklis, Sanjeev Galande, Shubha Tole

**Affiliations:** ^1^Department of Biological Sciences, Tata Institute of Fundamental Research, Mumbai 400005, India,; ^2^Indian Institute of Science, Education, and Research, Pune 411008, India,; ^3^Symbiosis School of Biomedical Sciences, Symbiosis International University, Lavale, Pune,; ^4^Department of Stem Cell and Regenerative Biology and; ^5^Center for Brain Science, Harvard Stem Cell Institute, Harvard University, Cambridge, Massachusetts 02138

**Keywords:** cell fate, chromatin, epigenetics, lamination, progenitor, specification

## Abstract

In the developing cerebral cortex, sequential transcriptional programs take neuroepithelial cells from proliferating progenitors to differentiated neurons with unique molecular identities. The regulatory changes that occur in the chromatin of the progenitors are not well understood. During deep layer neurogenesis, we show that transcription factor LHX2 binds to distal regulatory elements of *Fezf2* and *Sox11*, critical determinants of neuron subtype identity in the mouse neocortex. We demonstrate that LHX2 binds to the nucleosome remodeling and histone deacetylase histone remodeling complex subunits LSD1, HDAC2, and RBBP4, which are proximal regulators of the epigenetic state of chromatin. When LHX2 is absent, active histone marks at the *Fezf2* and *Sox11* loci are increased. Loss of LHX2 produces an increase, and overexpression of LHX2 causes a decrease, in layer 5 *Fezf2* and CTIP2-expressing neurons. Our results provide mechanistic insight into how LHX2 acts as a necessary and sufficient regulator of genes that control cortical neuronal subtype identity.

**SIGNIFICANCE STATEMENT** The functional complexity of the cerebral cortex arises from an array of distinct neuronal subtypes with unique connectivity patterns that are produced from common progenitors. This study reveals that transcription factor LHX2 regulates the numbers of specific cortical output neuron subtypes by controlling the genes that are required to produce them. Loss or increase in LHX2 during neurogenesis is sufficient to increase or decrease, respectively, a particular subcerebrally projecting population. Mechanistically, LHX2 interacts with chromatin modifying protein complexes to edit the chromatin landscape of its targets *Fezf2* and *Sox11*, which regulates their expression and consequently the identities of the neurons produced. Thus, LHX2 is a key component of the control network for producing neurons that will participate in cortical circuitry.

## Introduction

The complex functions of the cerebral cortex arise from circuitry in which distinct neuronal subtypes serve specific functions. The diversity of cortical neurons arises from initially common progenitors in the ventricular zone. Work from several groups has identified a complex network of transcriptional controls for the specification, differentiation, diversity, and plasticity of neuronal subtype identity (Arlotta et al., 2005; Woodworth et al., 2012; Greig et al., 2013). FEZF2 is necessary for the specification of subcerebral projection neurons (SCPN), the dominant population of layer 5 output neurons. It is sufficient for SCPN generation from cortical progenitors during development *in vivo* (Chen et al., 2005; Molyneaux et al., 2005). Further, SOX4, SOX5, SOX11, and TBR1 are upstream regulators of *Fezf2* expression in the cerebral cortex, with the SOX factors binding at downstream enhancer element (E4) and TBR1 binding at 3′ noncoding region of *Fezf2* (Kwan et al., 2008; Lai et al., 2008; Bedogni et al., 2010; Han et al., 2011; McKenna et al., 2011; Shim et al., 2012). It is increasingly understood that dynamic control over timing and levels of expression of key regulators is required to determine the generation of distinct neuronal subtypes in the cortical plate: SCPN in layer 5 versus corticothalamic projection neurons (CThPN) in layer 6 (Lai et al., 2008; Tomassy et al., 2010; Cederquist et al., 2013; Greig et al., 2013). Control of gene expression involves regulation of the chromatin at the relevant loci. However, our understanding of the dynamic chromatin regulation of gene loci involved in neuronal subtype specification remains inadequate. Such understanding is required to address the fundamental open question of how overlapping transcription factors expressed in the ventricular zone are translated into instructions to produce precise sets of neuronal subtypes in a temporal sequence. Here, we report a previously unknown role for the LIM-homeodomain transcription factor LHX2, as a key regulator of *Fezf2* and *Sox11* that is well positioned to function early in the cascade of mechanisms that specify SCPN identity.

LHX2 has a fundamental role in early cortical specification, in which it acts as a cortical selector gene (Mangale et al., 2008). However, *Lhx2* continues to be expressed in the ventricular zone throughout the period of cortical neurogenesis and displays intriguing spatiotemporal dynamics (Bulchand et al., 2003). Whereas neurons of deep layers 5 and 6 rapidly repress *Lhx2* expression, superficial layer neurons continue to express LHX2 from their birth dates through maturity (Bulchand et al., 2003). We hypothesized that the dynamic regulation of *Lhx2* might be important for the production of particular neuronal subtypes that predominate in deep versus superficial layers.

In this paper, we report the first evidence that LHX2 regulates cortical neuronal subtype identity. Loss of LHX2 function causes a striking increase in the layer 5 neurons expressing high levels of *Fezf2* and CTIP2, indicating SCPN fate (Arlotta et al., 2005; Molyneaux et al., 2005). It also causes a reduction in layer 6 neurons expressing TBR1 (predominantly CThPN fate) (Hevner et al., 2001; McKenna et al., 2011). We performed chromatin immunoprecipitation followed by sequencing (ChIP-Seq), and identified LHX2 occupancy on distal regulatory elements associated with *Fezf2* and its regulator, *Sox11*. To elucidate the mechanisms by which LHX2 might regulate *Fezf2* and *Sox11*, we performed protein IP followed by mass spectrometry and identified members of the nucleosome remodeling and histone deacetylase (NuRD) complex of chromatin regulators to be binding partners of LHX2. The transcription start sites (TSSs) and the LHX2 binding sites of both *Fezf2* and *Sox11* are epigenetically modified in an LHX2-dependent manner and display histone marks corresponding to the activity of NuRD complex. Finally, we show that LHX2 overexpression causes a decrease in the high *Fezf2*/CTIP2^+^-expressing (SCPN) population. Together, these results demonstrate that LHX2 is both necessary and sufficient to regulate the numbers of deep layer 5 corticofugal projection neurons that express the *Fezf2*/CTIP2 signature and that it functions by modulating the epigenetic marks on key factors that control cortical neuron subtype identity.

## Materials and Methods

### 

#### 

##### Mice.

All animal protocols were approved by the Institutional Animal Ethics Committee (Tata Institute of Fundamental Research, Mumbai, India) according to regulations formulated by the Committee for the Purpose of Control and Supervision of Experiments on Animals, India. The floxed LIM homeobox2 (*Lhx2*) line (*Lhx2lox/lox*) and *Emx1Cre^YL^* lines used in this study have been described previously by Mangale et al. (2008). The *Emx1Cre^YL^*(Jin et al., 2000) was obtained as a gift from Prof. Yuqing Li at University of Florida College of Medicine. The floxed *Lhx2* line was a gift from Prof. Edwin Monuki at the University of California, Irvine.

Timed pregnant female mice were obtained from the Tata Institute animal breeding facility, and embryos of both sexes were used for the experiments. Noon of the day the vaginal plug was observed was considered embryonic day (E) 0.5. Early-age embryos were staged by somite number and genotyped using PCR. Animals were genotyped and assigned to groups accordingly. Controls used for each experiment were age-matched littermates.

##### ChIP sequencing.

For each ChIP sequencing experiment, 50 μg chromatin and 4 μg antibody were used per IP. To obtain chromatin, brains from E12.5 embryos were harvested and the neocortical tissue was isolated in cold 0.5% glucose in PBS with 1× Protease inhibitor mixture (Sigma). The tissue was cross-linked immediately after harvesting with 1% formaldehyde (Thermo Scientific). Chromatin was sonicated using a Covaris S220 sonicator for 18 cycles of 60 s ON and 30 s OFF (5% Duty cycle, 2 Intensity and 200 cycles per burst) to get chromatin within the size range of 100–500 bp. The following antibodies were used for ChIP: goat α-LHX2 (Santa Cruz Biotechnology SC19344), goat IgG (Bangalore Genei). The protein-DNA complex was pulled down using Protein A-G magnetic beads (Dynabeads, Invitrogen). The immunoprecipitated DNA was purified using phenol-chloroform-isoamyl alcohol (Ambion). Sequencing libraries were prepared using SOLiD ChIP-Seq library preparation kit, and sequencing was performed on the SOLiD 4 System (Applied Biosystems). Five bases each were trimmed on the 5′ and 3′ ends, and reads were aligned to the reference genome mm^9^ using bowtie 1. Peaks were called using the MACS 1.4 program with default settings. The UCSC browser was used for data visualization.

##### ChIP-qPCR.

In each ChIP-qPCR experiment for validation of binding of LHX2 and/or NURD complex protein members on LHX2 binding regions and TSS, 10 μg chromatin and 2 μg antibody was used per IP. For each Histone mark ChIP-qPCR, 5 μg chromatin and 1 μg antibody was used per IP. The following antibodies were used for ChIP: goat α-LHX2 (Santa Cruz- SC19344), goat IgG (Bangalore Genei), rabbit anti-Kdm1a/LSD1 (Abcam, #ab17721), rabbit anti-HDAC2 (Abcam, #ab7029), rabbit anti-RBBP4 (Abcam, #ab38135), rabbit anti IgG (as control IgG for NuRD complex protein ChIPs) (Sigma, #18140), anti-Histone H3 antibody (ab1791), rabbit anti-H3K4me3 (Diagenode, C15410030), rabbit anti-H3K9ac (Diagenode, C15410004).

ChIP was performed as described above. For LHX2 and NURD complex protein members, individual enrichment over the control genomic region was assessed by performing ChIP-qPCR with primers specific for these regions using the SYBR Green master mix (Roche). For histone marks, ChIP signals for H3K4me3 and H3K9ac were normalized to total H3. ChIP-qPCRs were done in duplicates, and at least three independent experiments were performed for each ChIP-qPCR. For statistical analysis, independent experiments were used to calculate average, SEM, and significance value.

Information of the primers used for ChIP-qPCR is given (5′ to 3′) as follows: LHX2 BR on *Fezf2*: forward, TAGGCATGGAACGCAATGTA; reverse, TGGGACAGGAAGAAAAGACG; TSS *Fezf2*: forward, CCCTGGTGTCCGTCTAATCA; reverse, CGCCACATCCTAATGAGGTAA; LHX2 BR on *Sox11*: forward, GCAGACACAGCCGTCCAT; reverse, GGAACAATACACGGGTCTCC; TSS *Sox11*: forward, CACTACTCCCACCAGCCAAT; reverse, GCACTCGCGGATTTCTTTT; and control genomic region: forward, GGGTCACTGAGGCAAAAATC; reverse, GCCTATCACCTGCAGGATTC.

##### IP and Western blotting: mass spectrometry.

For mass spectrometric analysis of the LHX2 interacting proteins, IP was performed using 10 mg of protein sample and 20 μg of antibody. Brains from E15 embryos were harvested in cold 0.5% glucose in PBS with 1× Protease inhibitor mixture. For IP, samples were lysed in TNN buffer (50 mm Tris-Cl pH 7.5, 150 mm NaCl, 0.9% NP-40, 1 mm PMSF, 1× protease inhibitor mixture, and 1× phosphatase inhibitor mixture) using a Dounce homogenizer. The lysate was centrifuged at 13,000 rpm for 45 min at 4°C to remove membranes and other debris. Supernatant was collected. Goat anti-LHX2 (Santa Cruz Biotechnology, SC 19344) was used for LHX2 pulldown, and goat IgG (Bangalore Genei) was used as the negative control. The magnetic beads used for IP along with the precipitated proteins were resuspended in Laemmli buffer (63 mm Tris-HCl, 2% SDS, 0.0025% bromophenol blue, 10% glycerol) without β-mercaptoethanol and boiled at 65°C for 5 min. Proteins obtained by IP were resolved on 8% SDS gel of 1 mm thickness. The gel was processed for silver staining, and in-gel digestion was done according to the protocol described by Shevchenko et al. (1996). Mass spectrometric analysis was done at the Mass Spectrometry Facility, Tata Institute of Fundamental Research. Three biological replicates of LHX2-IP material were subjected to mass spectrometric analysis, and individual candidates were validated using IP. β-Mercaptoethanol was not included in the Laemmli buffer; therefore, the proteins tend to run at molecular weights higher than expected, which is reflected in our data.

##### LHX2 IPs and reverse IPs.

For the LHX2 IPs and reverse IPs (with subunits of the NURD complex), the IP was performed using 500 μg of lysate and 1 μg of antibody. For the LHX2 IPs, the same antibodies were used that have been previously mentioned for mass spectrometry. For the reverse IPs, the following antibodies were used: rabbit anti-KDM1A/LSD1 (Abcam, #ab17721), rabbit anti-HDAC2 (Abcam, #ab7029), rabbit anti-RBBP4 (Abcam, #ab38135), and rabbit anti-IgG (Sigma, #18140), which was used as a control for all the reverse IPs. IPs were performed as previously described for mass spectrometric analysis. The magnetic beads used for IP along with the precipitated proteins were resuspended in Laemmli buffer (63 mm Tris-HCl, 2% SDS, 0.0025% bromophenol blue, 10% glycerol) without β-mercaptoethanol and boiled at 65°C for 5 min for all the IPs other than the LHX2 IP probed with LSD1. LSD1 has a high molecular weight, which makes the visualization of the band difficult in the IP lanes as IgG produces a dark band at higher molecular weights. For this particular IP, the beads were resuspended in Laemmli buffer with β-mercaptoethanol and boiled at 95°C for 3 min to degrade the IgG, which resulted in a band at ∼55 kDa, thus making the band corresponding to LSD1 clearly visible. The resuspended samples were run on SDS-containing gel and then transferred on a PVDF membrane (Roche) in transfer buffer containing 20% methanol and 0.01% SDS at 90 V for 100 min. LHX2 affinity purified mouse monoclonal antibody, custom made from Bioclone, was used at a dilution of 1:1000 for probing the blots for the LHX2 IPs and reverse IPs. The monoclonal antibody was validated before use. The aforementioned antibodies (anti-KDM1a, anti-HDAC2, and anti-RBBP4) were used for probing the Western blots at a dilution of 1:1000. Blots were developed using ECL substrate (GE Healthcare). Three biological replicates for each IP were performed.

##### ISH.

Digoxigenin-labeled RNA probes were used for ISH. Digoxigenin-labeled NTPs were obtained from Roche and used to make riboprobes. Brains were sectioned (30 μm) using a freezing microtome. The sections were mounted on Superfrost Plus slides (Erie Scientific). After fixing in 4% (w/v) PFA, sections were washed with 1× PBS. The sections were then treated with Proteinase K in TE buffer (1 μg/ml). Postfixation was done using 4% PFA, and the sections were washed with 1× PBS. The sections were hybridized for 16 h at 70°C in buffer containing 50% (v/v) formamide, 5× SSC and 1% (w/v) SDS. Stringent washes posthybridization were performed with Solution X (50% formamide, 2× SSC, and 1% SDS) followed by 2× SSC and then 0.2× SSC. Overnight incubation at 4°C with anti-digoxigenin antibody tagged with alkaline phosphatase (1:5000, Roche, catalog #12486523). Antibody was detected using substrate NBT/BCIP (Roche, 4-nitroblue tetrazolium chloride, catalog #70210625; 5-bromo-4-chloro-3-idolyl phosphate, catalog #70251721). Slides were counterstained with Fast Red (Sigma N3020), coverslipped using DPX mountant, and imaged. ISH for each marker was performed in at least four biological replicates.

Plasmids used for generating probes were obtained from Jeffrey Macklis, Harvard University (*Fezf2*); Susan McConnell, Stanford University (*ER81*); Anastassia Stoykova, University of Göttingen (Id2); Edwin Monuki, University of California, Irvine (*Cux2*); Robert Hevner, University of Washington (*Tbr1*); and Cliff Ragsdale, University of Chicago (Rorβ). Probes for *Lhx2* and *Sox11* were generated using PCR primers, the information for which is given (5′ to 3′) as follows: *Lhx2*: forward, GATGTAGCTGCCCCCACGCC; reverse, TGTGGAACAGCATCGCGGC; *Sox11*: forward, CGCTGGAAGATGCTGAAGGA; reverse, CCAGCGACAGGGATAGGTTC.

##### Immunohistochemistry.

**Figure 1.**
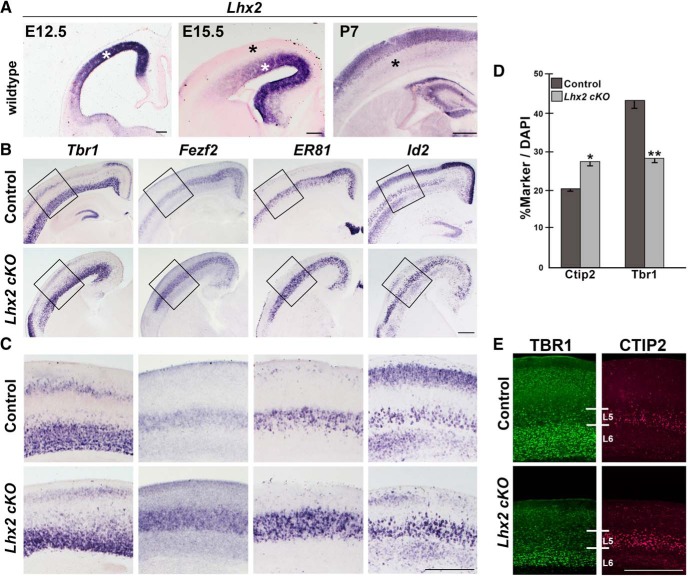
Cortex-specific loss of *Lhx2* alters the expression of neuronal subtype markers in layers 5 and 6. ***A***, Expression of *Lhx2* in control brains is seen in the ventricular zone at E12.5 and E15.5 (white asterisks) but not in postmitotic deep layer neurons at E15.5 and P7 (black asterisks). ***B***, ***C***, Expression of neuronal subtype markers at P7 in control and *LHX2cKO* brains reveals a decrease in the *Tbr1*-expressing population and an increase in the *Fezf2*, *ER81*, and *Id2*-expressing population. ***B***, Boxed regions are shown in ***C***. ***D***, ***E***, The numbers of cells expressing TBR1 or CTIP2, expressed as a percentage of all DAPI-stained cells in layer 5 (L5) + layer 6 (L6). Error bars indicate SEM. Scale bars, 500 μm. *p < 0.05. **p < 0.001.

Primary antibodies used were as follows: biotinylated goat anti-GFP (1:400; Abcam, catalog #ab6658), rabbit anti-TBR1 (1:200; Abcam, catalog #ab31940), rat anti-CTIP2 (1:200; Abcam, catalog #ab18465), and mouse anti-SATB2 (1:200; Abcam, catalog #ab51502). Secondary antibodies used were as follows: streptavidin Alexa-488 (1:800; Invitrogen, catalog #S32354) for GFP. Goat anti-rabbit antibody conjugated to Alexa-488 (1:400, Molecular Probes, catalog #A11008) for TBR1. Goat anti-rat antibody conjugated to Alexa-568 (1:400, Molecular Probes, catalog #A11077) for CTIP2. Goat anti-mouse antibody conjugated to Alexa-647 (1:400, Molecular Probes, catalog #A21235) for SATB2. Tissue processing for immunohistochemistry was performed as described by Subramanian et al. (2011). For each control and experimental condition, ≥100 cells were counted from each of five biological replicates for Figure 1 and from each of three biological replicates for Figure 5.

##### *In utero* electroporation.

All procedures conducted followed the guidelines prescribed by the Institutional Animal Ethics Committee. Swiss mice obtained from the Tata Institute of Fundamental Research animal breeding facility were used for electroporation. E12.5 timed pregnant mice were anesthetized using isoflurane (Forane, Abbott India). The surgical procedure performed has previously been described by Subramanian et al. (2011). The 3–4 μl plasmid DNA of concentration ∼2 μg/μl dissolved in nuclease free water and mixed with Fast Green dye was injected into the lateral ventricle of the embryos using a fine-glass microcapillary. For electroporation, a BTX CUY21 electroporator [32 V (E12.5), 4 pulses, 50 ms pulse length, ∼1.0 s pulse interval was used. Electric pulses were delivered using 3 mm paddle electrodes. The cortex was targeted by placing the positive electrode on the side of the dorsal wall of the lateral ventricle. The uterine horns were replaced, and the incision was sewn with surgical sutures. Animals were kept on a 37°C warm plate for half an hour for postsurgical recovery. An oral suspension of Meloxicam (Melonex, United Pharmacies) was mixed with the water in the feeding bottles of the dams (0.6 μl/ml) as an analgesic and given to the animals until 2 d after surgery. DNA constructs *Lhx2-GFP* and *pCAGIRES2-EGFP* were used as described by Subramanian et al. (2011).

##### Imaging.

Bright-field images were taken using a Zeiss Axioplan 2 + microscope, Nikon Digital Sight DS-F12 camera, and Nikon NIS 4.0 imaging software. Images of immunohistochemistry were obtained using a Zeiss LSM 5 Exciter-AxioImager M1 imaging system and Zeiss LSM510 imaging system. Image stacks were generated by scanning at intervals of 4 μm for lower magnification and at intervals of 0.8 μm for higher magnification using filters of the appropriate wavelengths. The stacks were analyzed, merged, and projected using ImageJ software from the National Institutes of Health. Figure panels were prepared using Adobe Photoshop CS6.

##### Statistical analysis.

Statistical analysis was performed using the unpaired two-tailed Student's *t* test. Error bars indicate SEM. All primary data from immunohistochemistry and ISH experiments were analyzed by one investigator and then confirmed by a second, independent investigator.

## Results

*Lhx2* is strongly expressed in the ventricular zone at embryonic day (E) 12.5 and E15.5 (Fig. 1*A*, white asterisks) but is not detectable in postmitotic deep layer neurons accumulated in the cortical plate from E15.5 onwards. By postnatal stages, deep layer neurons do not detectably express *Lhx2* (Fig. 1*A*, black asterisks). We generated cortex-specific *Lhx2* conditional mutant brains using *Emx1Cre^YL^*, which we have previously characterized as a reagent that achieves near-complete recombination in the dorsal telencephalon by E11.5, a day later than the commonly used *Emx1Cre^KJ^* line (Shetty et al., 2013). The *Emx1Cre^YL^* line is crucial to the present study because it spares the neocortex (Shetty et al., 2013); removing LHX2 from the dorsal telencephalon before E11.5 using *Emx1Cre^KJ^* causes a transformation of neocortex to paleocortex (Chou et al., 2009). For the rest of this study, *Emx1Cre^YL^* will be referred to as simply “*Emx1Cre*,” and the resulting brains will be referred to as “cortex-specific conditional mutant brains.”

In cortex-specific *Lhx2* conditional mutant mice (*Emx1Cre^YL^*) (Shetty et al., 2013), as well as in the pan-CNS *Lhx2* conditional mutant mice (*Nestin Cre*) (Chou and O'Leary, 2013), the cortex is thinner than in control brains. Although all layers are specified, the later born superficial layers are considerably thinner in mutant brains compared with controls. In these studies, the deep layers 5 and 6 did not appear to be as drastically affected (Chou and O'Leary, 2013; Shetty et al., 2013). In the present study, we discovered a previously unreported phenotype in the deep layers 5 and 6 of cortex-specific *Lhx2* conditional mutant brains.

On close examination of postnatal day (P) 7 brains, we found that *Tbr1*-expressing layer 6 neurons are reduced in *Lhx2* conditional mutant brains compared with controls. In contrast, layer 5 is substantially expanded as revealed by the expression of *ER81*, *Id2*, high-level *Fezf2*, and CTIP2, a downstream effector of FEZF2 (Fig. 1*B*,*C*,*E*) (Arlotta et al., 2005; Molyneaux et al., 2005; Chen et al., 2008; Rouaux and Arlotta, 2013). Cell counts of CTIP2-expressing and TBR1-expressing cells as a percentage of the total DAPI-stained population (layers 5 + 6) confirms the increase (CTIP2) and decrease (TBR1) in the respective populations (Fig. 1*D*,*E*). This suggested that LHX2 may regulate neuronal subtype fate specification.

**Figure 2.**
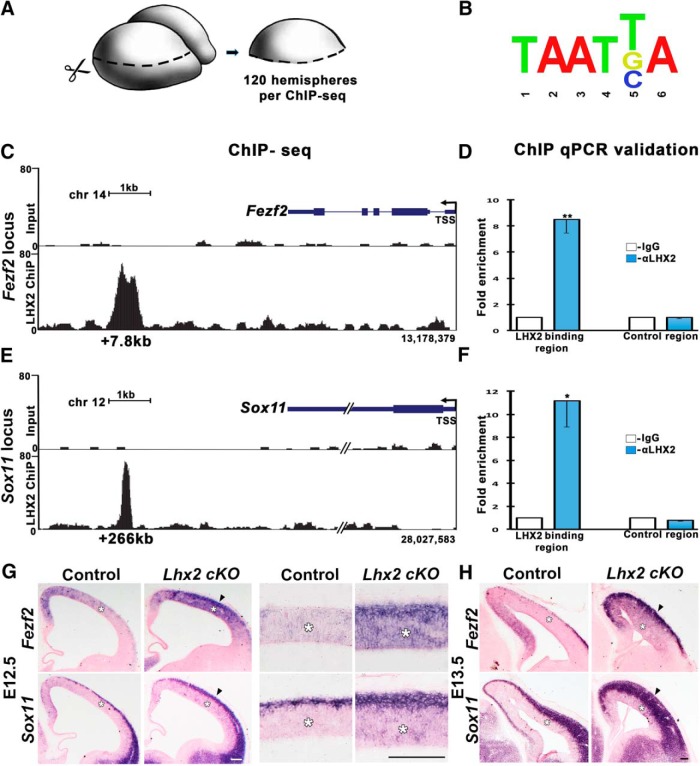
LHX2 occupies enhancer elements of cortical neuron subtype regulators *Fezf2* and *Sox11*. ***A***, Diagram illustrating dissection of cortical primordia. ***B***, The LHX2 binding site sequence reported in the literature was also found in the LHX2 binding regions of *Fezf2* and *Sox11*. ***C–F***, ChIP-Seq data showing UCSC genome browser tracks of the LHX2 occupancy profile at the *Fezf2* (***C***) and the *Sox11* (***E***) loci. Each LHX2-binding region was validated by ChIP followed by qPCR analysis (***D***,***F***). ***G***, ***H***, Examination of *Fezf2* and *Sox11* at E12.5 (***G***), 1 d after cortex-specific loss of LHX2, reveals an increased expression of *Fezf2* and *Sox11* in the ventricular zone (white asterisks) and an increased accumulation of *Fezf2*- and *Sox11*-expressing postmitotic cells (arrowhead). High-magnification images are displayed alongside. ***H***, By E13.5, the increase in the ventricular zone has attenuated (white asterisks), but the expression in the cortical plate has expanded (arrowhead). Error bars indicate SEM. Scale bar, 100 μm. *p < 0.05. **p < 0.001.

To investigate this possibility, we harvested cortical primordia from embryonic telencephalic hemispheres at E12.5, and performed ChIP using anti-LHX2 antibody, followed by sequencing (ChIP-Seq; Fig. 2*A*). We screened the loci associated with LHX2 binding regions for known regulators of cortical neuronal subtype identity. Two candidates, *Fezf2* and its known transcriptional regulator *Sox11* (Shim et al., 2012), displayed LHX2 occupancy on putative distal regulatory regions.

During deep layer neurogenesis, both these factors promote layer 5 SCPN fate, and FEZF2 represses layer 6 TBR1^+^ CThPN fate (Molyneaux et al., 2005; Han et al., 2011; McKenna et al., 2011). The LHX2 occupancy peaks for the *Fezf2* and *Sox11* loci were at positions distant from the TSS, 7.8 and 266 kb, respectively (Fig. 2*C*,*E*). These regions each contained an LHX2 binding site sequence reported in the literature (Berger et al., 2008; Wilson et al., 2008) (Fig. 2*B*). The LHX2 occupancy was further confirmed by ChIP-qPCR using primers designed for the specific binding region (Fig. 2*D*,*F*; for details, see Materials and Methods).

To test whether LHX2 expression in the ventricular zone regulates the transcription of *Fezf2* and *Sox11*, we examined their expression in E12.5 control and *Lhx2* cortex-specific conditional mutant brains (Fig. 2*G*). The *Emx1Cre^YL^* driver causes widespread recombination in the cortical primordium by E11.5 (Shetty et al., 2013).By E12.5, there was a striking upregulation of *Fezf2* in the ventricular zone in *LHX2cKO* brains compared with controls. *Sox11*, normally only seen in postmitotic neurons, also displayed a modest increase in the *LHX2cKO* ventricular zone. In addition, both *Fezf2*- and *Sox11*-expressing cells were increased in number in the cortical plate of *LHX2cKO* brains (Fig. 2*G*), which is consistent with the increased numbers of *Fezf2*/CTIP2-expressing cells seen postnatally (Fig. 1). This effect of increased expression in the cortical plate was even more striking at E13.5, by which stage the *Fezf2/Sox11* upregulation in the ventricular zone has attenuated (Fig. 2*H*). This suggests that LHX2-mediated repression of *Fezf2* and *Sox11* is particularly important in the E11.5–12.5 ventricular zone, corresponding to the peak of layer 6/5 neurogenesis. The continued effects of loss of *Lhx2* are seen when the postmitotic neurons reach the cortical plate by E13.5. Therefore, these data reveal that LHX2 binds distal regulatory elements of *Fezf2* and *Sox11*, and loss of LHX2 results in upregulation of these genes in the cortical ventricular zone during the time of peak production of SCPNs.

**Figure 3.**
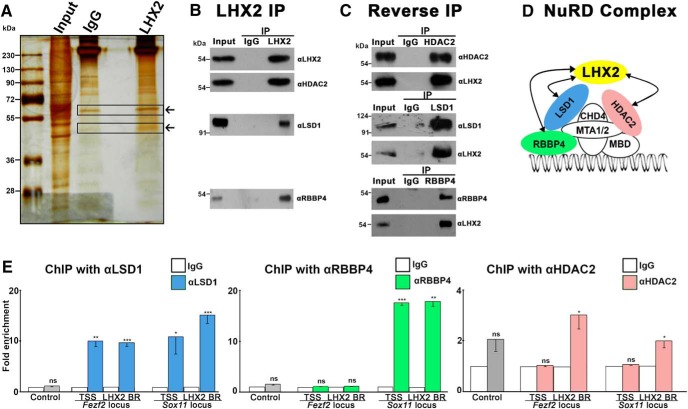
LHX2 binds specific subunits of the NuRD complex. ***A***, A silver-stained gel showing control (IgG) and LHX2 IP from cortical tissue. Boxes represent bands that were excised and from which the proteins were analyzed using mass spectrometry. ***B***, Putative LHX2 binding partners identified in the mass spectrometry (HDAC2, LSD1, and RBBP4) were validated by Western blot (immunoblot) analysis of LHX2 IP material using antibodies against each candidate partner. ***C***, Reverse validation of interactions was performed by performing IP for each binding partner and probing the Western blot using anti-LHX2. ***D***, Diagram illustrating the NuRD complex and members that bind LHX2. ***E***, ChIP-qPCR-based occupancy analysis demonstrating the binding of LSD1, HDAC2, and RBBP4 to either the TSS and/or the LHX2 binding region (LHX2 BR) on *Fezf2* and/or *Sox11. y*-axis indicates fold enrichment over IgG at the respective loci. CHD4, Chromodomain helicase DNA binding protein 4; HDAC2, histone deacetylase 2; LSD1, lysine-specific histone demethylase1; MDB, methyl CpG binding domain protein; MTA1/2, metastasis-associated protein1/2; RBBP4, retinoblastoma binding protein 4. ***A***, The image of the silver-stained gel has been cropped to remove the lanes corresponding “unbound” fractions of control IgG and LHX2 IP that were on the left of the marker lane. ***B***, ***C***, The Western blots are cropped from full-length Western blots. The original uncropped images are available upon request. Error bars indicate SEM. *p < 0.05. **p < 0.001. ***p < 0.0001.

Transcription factors usually act in multimeric complexes to achieve their functions (Vernimmen and Bickmore, 2015). To identify binding partners of LHX2, we performed IP from cortical tissue using anti-LHX2 antibody, and ran the resulting samples on an SDS-PAGE gel. Silver staining revealed bands that were differentially enriched in comparison with a control IgG IP (Fig. 3*A*). These were excised and the proteins identified using mass spectrometry. Mass spectrometry was performed for 2 additional biological replicates of LHX2-IP material, for which all bands were excised and sequenced. The resulting dataset was manually curated for known chromatin modifiers. Three subunits associated with the NuRD complex, LSD1, RBBP4, and HDAC2 (Wang et al., 2009), were identified as potential binding partners of LHX2. These were individually validated in three biological replicates, by performing LHX2 IP followed by Western blotting using antibodies against the specific NuRD complex proteins (Fig. 3*B*). Reverse validations were also performed, in which LSD1/RBBP4/HDAC2-specific antibodies were used for IP, and the Western blot was probed with anti-LHX2 (Fig. 3*C*). Of the known components of the NuRD complex, the subunits that bind LHX2 are shown in color (Fig. 3*D*). Finally, we tested whether these particular subunits bind the same regions as LHX2 on the *Fezf2* and *Sox11* loci. We also tested the TSSs of both these genes. ChIP using anti-LSD1/anti-RBBP4/anti-HDAC2, followed by qPCR, revealed that all three NuRD complex subunits bind either the TSS and/or the LHX2 binding region (LHX2 BR) of *Fezf2* and/or *Sox11*. In summary, these data reveal that LHX2 binds to specific chromatin-regulatory factors that also bind the distal LHX2 occupancy sites and/or the TSS of key neuronal subtype identity regulators *Fezf2* and *Sox11*.

**Figure 4.**
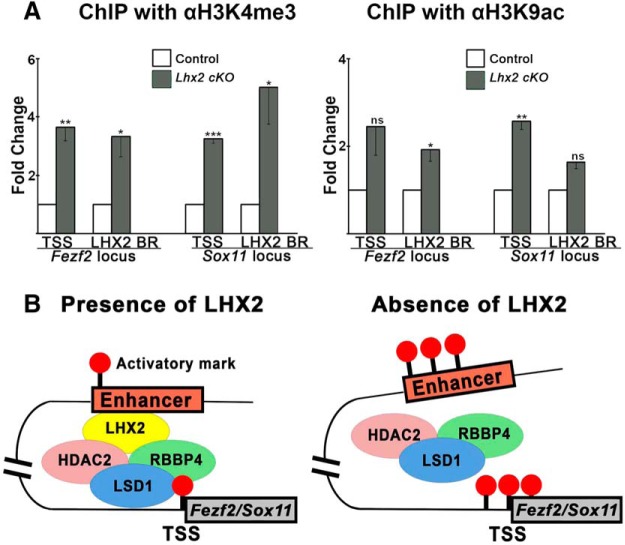
Loss of LHX2 causes an increase in active epigenetic histone marks on *Fezf2* and *Sox11*. ***A***, ChIP-qPCR for active histone marks H3K4me3 and H3K9ac in E12.5 control versus *LHX2cKO* cortical tissue. *y*-axis indicates fold change over control at the respective loci. ***B***, Diagram depicting proposed model of chromatin looping bringing distant regulatory elements and TSS near each other. We propose that LHX2 binding to the regulatory elements of its target genes *Fezf2* and *Sox11* recruits the NuRD subunits LSD1 and HDAC2, which associate with the TSS and the LHX2 binding region, leading to erasure of active marks. In the absence of LHX2, the active marks are enriched. Error bars indicate SEM. *p < 0.05. **p < 0.001. ***p < 0.0001.

Epigenetic marks on the distal regulatory elements and the TSSs are reflective of the transcription status of the gene, and understanding the factors that bring this about gives a mechanistic insight into the process. We examined whether epigenetic marks on the TSS and the LHX2 binding regions of *Fezf2* and *Sox11* were altered in *Lhx2* mutant tissue. We harvested E12.5 tissue from control and *LHX2cKO* cortices and performed ChIP using antibodies against two well-established active marks, H3K4me3 and H3K9Ac (Karmodiya et al., 2012). We found both these marks to be significantly enriched at the TSS and/or LHX2 BR of both *Fezf2* and *Sox11* in *LHX2cKO* tissue (Fig. 4*A*). Therefore, the presence of LHX2 is essential to erase these marks at these sites. Because LHX2 binds a region distant from the TSS, its regulation of active marks at both sites may be explained by a chromatin looping model in which the putative enhancer and the TSSs are brought near each other in the presence of LHX2. Such a model would permit chromatin-modifying machinery, including, but not limited to, the proteins we identified, to erase the active marks. In the absence of LHX2, this function may be diminished, and the active marks are enriched (Fig. 4*B*), thereby leading to an aberrant expression of *Fezf2* and *Sox11*.

**Figure 5.**
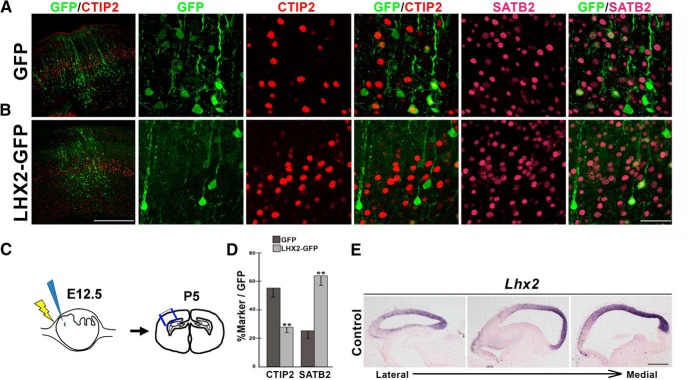
LHX2 is necessary and sufficient to regulate molecular subtype identity. ***A***, ***B***, Electroporation of control *GFP* (***A***) or *LHX2-GFP* (***B***) at E12.5 and examination at P5 reveal GFP-expressing cells in the region of electroporation, overlapping with CTIP2-expressing layer 5 cells. Individual high-magnification confocal images of GFP, CTIP2, SATB2, and the corresponding merged images of GFP/CTIP2 and GFP/SATB2 are shown alongside the low-magnification GFP/CTIP2 image. ***C***, Diagram illustrating *in utero* electroporation at E12.5, and examination of brain sections at P5. ***D***, The percentage of electroporated (GFP-expressing) cells that also express CTIP2 or SATB2 reveals a striking decrease in CTIP2-expressing cells and an increase in SATB2-expressing cells upon electroporation of *LHX2-GFP*. ***E***, A gradient of *Lhx2* expression is seen in a series of sagittal sections at E12.5. Scale bars: ***A***, ***B***, 500 μm (low-magnification images), 50 μm (high-magnification images); ***E***, 500 μm. Error bars indicate SEM. **p < 0.001.

Loss of LHX2 results in increased activation/expression of target genes, and correlated with this, an increase in the number of layer 5 *Fezf2*/CTIP2^+^ neurons. We tested whether overexpression of *Lhx2* would have the opposite effect of reducing the numbers of these neurons. We used an *LHX2-GFP* construct for *in utero* electroporation at E12.5 (Fig. 5*A*,*B*). The quantifications demonstrate that LHX2 overexpression causes a 50% decrease in CTIP2-expressing cells and a 59% increase in SATB2-positive cells in layer 5, consistent with a role for LHX2 in modulating relative numbers of neurons with distinct molecular subtype signatures (Fig. 5*D*).

In summary, our results show that loss or overexpression of *Lhx2* regulates key genes that specify neuronal subtype identity. LHX2 appears to achieve this regulation by recruiting chromatin-modifying proteins, and loss of LHX2 causes changes in the epigenetic marks associated with its target genes. These findings position LHX2 as a critical component of the network of factors that control the production of an appropriate proportion of neuronal subtypes in the developing cerebral cortex.

## Discussion

We present an extensive mechanistic analysis of how LHX2, a transcription factor expressed in the cortical ventricular zone, can exert regulatory control on mechanisms that confer subtype identity to cortical neurons. *Emx1Cre* acts in the ventricular zone, and postmitotic neurons arising from these progenitors would also be expected to carry any alleles that undergo recombination as a result of the *Emx1Cre* action. Therefore, an obvious question is whether the effects of LHX2 removal are relevant in the ventricular zone progenitors themselves, or in the newly postmitotic neurons they produce. A recent study that used *NexCre* to delete LHX2 specifically in postmitotic neurons starting from E11 offers clarity on this issue (Zembrzycki et al., 2015). This study elegantly demonstrated that *NexCre* action is not seen in proliferating progenitors in the ventricular zone but is seen in newly postmitotic cells (Zembrzycki et al., 2015, their Figure S2) and that molecular specification of the different cortical layers is not affected (Zembrzycki et al., 2015, their Fig. 3). Therefore, the function of LHX2 we uncovered in the present study appears to be a novel role for LHX2 that operates in ventricular zone progenitors, the effects of which are seen by way of molecular dysregulation of its target genes in the ventricular zone itself, and also evident in altered proportions of cortical neuronal subtypes in maturity.

Loss of LHX2 in the cortical ventricular zone has been reported to cause premature neurogenesis, resulting in a thicker cortical plate during the early stages of corticogenesis and as a consequence, depletion of the progenitor pool resulting in thinner superficial layers (Chou et al., 2013). However, by postnatal stages the combined effect of these two opposing phenomena result in a cortex with diminished thickness compared with that of wild-type brains (Chou et al., 2013; Shetty et al., 2013). A defect involving premature neurogenesis would be expected to leave the earliest born layer 6 relatively unaffected and have a progressively greater effect on the later born layers due to progenitor depletion. Our present study uncovers a paradoxical phenotype, such that layer 6 is thinner than normal, and layer 5 is expanded compared with control brains (Fig. 1). This is not explained by a progressive depletion of the progenitor pool (Chou et al., 2013) but, rather, suggests an entirely different defect: that of neuronal subtype fate specification. Removal of LHX2 leads to upregulation of *Fezf2* in the ventricular zone, which is known to suppress *Tbr1* (Mckenna et al., 2011). The decrease in layer6 TBR1 neurons may not be a consequence of direct regulation by LHX2, but an indirect result of *Fezf2* upregulation in the absence of LHX2. Therefore, in addition to its previously described role in regulating progenitor proliferation, this multifunctional transcription factor also regulates neuronal subtype identity. This is the first report of such a function for LHX2.

To provide a comprehensive understanding of the mechanisms at play, studies of gene regulation should include both an examination of the protein complexes in which the regulatory molecule acts, as well as its occupancy on target gene loci. We report a novel molecular role for LHX2, in which it partners with proteins HDAC2, LSD1, and RBBP4, that are found together in the NuRD-HDAC complex. However, these proteins display differential occupancy on *Fezf2* and *Sox11* in that RBBP4 does not appear to bind the *Fezf2* locus on either the TSS or the LHX2 BR, whereas LSD1 binds both these regions on both *Fezf2* and *Sox11*, and HDAC2 does so only at the LHX2 BR on both loci. This raises the possibility that may not act together within the holo-NuRD complex but may act independently or in other combinations in a site-specific context.

LSD1 can be associated with the NuRD complex (Wang et al., 2009) or also the CoREST repressor complex (Yang et al., 2006; Wang et al., 2007). RBBP4 is associated with the Polycomb repressive complex (PRC2) (Kuzmichev et al., 2002). Thus, LHX2 may associate with specific chromatin modifiers in a context-dependent manner. Exploring the changing nature of such associations will give new insights to the temporally dynamic controls on cell fate specification.

Furthermore, we demonstrate that there are functional consequences within a day of loss of LHX2. These include an increase in the H3K9Ac and H3K4Me3 epigenetic marks that are associated with actively transcribed genes (Karmodiya et al., 2012) as well as an abnormal overexpression of the target genes in the ventricular zone by E12.5, an effect that attenuates by E13.5. Layer 5 neurons are produced during this time window, and there is a corresponding increase in the *Fezf2*/CTIP2^+^-expressing layer 5 SCPN-like neurons seen at postnatal stages.

As a final test of LHX2 function, overexpression of *Lhx2* at E12.5 has the opposite effect of dramatically reducing the number of CTIP2-expressing neurons. Given the normally high levels of *Lhx2* in the cortical primordium, the effects of *Lhx2* overexpression may arise due to its continued expression in postmitotic deep layer neurons in which it is normally not expressed. This suggests that LHX2 may be able to epigenetically silence its targets in postmitotic neurons as well. Although additional work is needed to understand how *Lhx2* fits in with the other known regulators of *Fezf2*, our study offers new directions and insight on how this question may be explored further in progenitors and postmitotic neurons.

Of the many roles reported for LHX2 in the literature, this is perhaps the most surprising, given that *Lhx2* appears to be expressed in all progenitors at E10.5-E11.5 (Rincón-Limas et al., 1999; Lu et al., 2000) and displays continued expression in a gradient throughout the period of cortical neurogenesis (Bulchand et al., 2003). At E12.5, *Lhx2* expression displays a gradient in the cortical primordium, from caudomedial^high^ to rostrolateral^low^ (Nakagawa et al., 1999) (Fig. 5*E*). The fact that *Lhx2* overexpression at E12.5 is able to dramatically reduce the number of postnatal CTIP2 expressing neurons in layer 5, and loss of *Lhx2* is able to increase them, indicates that levels of *Lhx2* in the E12.5 neuroepithelium may play an important role in governing the neuronal number corresponding to this population, together with other regulators across the rostrocaudal axis. Work from several groups has reported that regulators of neuronal subtype identity, such as *Fezf2*, *COUP-TF1*, *CB1R*, and *Cux2*, display widespread to limited expression in the ventricular zone (Hirata et al., 2004; Nieto et al., 2004; Zimmer et al., 2004; Chen et al., 2005; Molyneaux et al., 2005; Faedo et al., 2008; Cubelos et al., 2010; Tomassy et al., 2010; Díaz-Alonso et al., 2012, 2015; Rodríguez-Tornos et al., 2016). Of these, *Fezf2* is expressed widely in the ventricular zone at E12.5 but is seen only in postmitotic neurons by E15.5 (Hirata et al., 2004), and neurons in all cortical layers appear to originate from FEZF2^+^ lineage (Guo et al., 2013). Although this immediately suggests a strong parallel with the temporal fate specification model that has been elegantly described in *Drosophila* neuroblasts (Kohwi and Doe, 2013), the function of ventricular *Fezf2*, if any, in determining postmitotic identity of subcerebral projection neurons remains to be elucidated, and our finding that loss of LHX2 results in an upregulation of *Fezf2* in the ventricular zone itself adds further motivation to exploring this question.

Our study motivates a mechanistic analysis of the transcription factor network that controls cortical neuronal subtype identity, from an epigenetic angle. It is important to unravel what terms, such as induction or suppression, actually entail at the level of the chromatin. Such an analysis may offer some clarity on why some genes appear more responsive to regulatory controls (e.g., whether they are in an epigenetically “poised” state), with both active and repressive marks, in which case they can be induced or suppressed with short time delays (Bernstein et al., 2006; Mikkelsen et al., 2007; Hirabayashi and Gotoh, 2010). In some cases, transcription factors may compete for or sequester key components of chromatin remodeling complexes and cause regulation of target genes in this fashion. One example of this is the regulation of *Ctip2* by SATB2 and LMO4 (Harb et al., 2016). This study elegantly demonstrates that LMO4 binds and sequesters HDAC1 and prevents it from participating in the SATB2-NuRD complex that normally suppresses *Ctip2*. In addition to the protein complexes that bind chromatin, distal enhancers associated with any target gene locus are equally important. For example, Shim et al. (2012) identified a region 7.3 kb downstream of the *Fezf2* TSS termed the E4 enhancer, which is the region at which activators of *Fezf2*, SOX4 and SOX11, compete for binding with repressor SOX5. SATB2 also binds this enhancer and positively regulates the expression of *Fezf2* in cortical neurons (McKenna et al., 2015). This enhancer is most strongly expressed in cortical progenitors and possibly drives the expression of FEZF2 in these cells (Eckler et al., 2014). How the activation or repression is achieved via this distal regulatory element will almost certainly be a fascinating story of epigenetic regulation of *Fezf2*. Moreover, in a role strikingly similar to that of LHX2, a recent study demonstrated that transcription factor CTIP1, expressed by newly postmitotic neurons, functions to specify SCPN versus CThPN identity in the cortex (Woodworth et al., 2016). It is in such a context that our results are of immediate interest because we position LHX2 among the known regulators of neuronal subtype identity that are expressed in the ventricular zone, the interactions of which are likely to determine area specific complements of deep layer neuronal subtypes along the rostrocaudal and mediolateral axes of the developing cortex.
